# Managing Cancer And Living Meaningfully: study protocol for a randomized controlled trial

**DOI:** 10.1186/s13063-015-0811-1

**Published:** 2015-09-03

**Authors:** Chris Lo, Sarah Hales, Anne Rydall, Tania Panday, Aubrey Chiu, Carmine Malfitano, Judy Jung, Madeline Li, Rinat Nissim, Camilla Zimmermann, Gary Rodin

**Affiliations:** Department of Supportive Care, Princess Margaret Cancer Centre, University Health Network, 16th Floor, 610 University Avenue, Toronto, ON M5G 2M9 Canada; Department of Psychiatry, University of Toronto, 8th Floor, 250 College Street, Toronto, ON M5T 1R8 Canada; Department of Medicine, University of Toronto, Suite RFE 3-805, 200 Elizabeth Street, Toronto, ON M5G 2C4 Canada

**Keywords:** Advanced cancer, Psychotherapy, Randomized controlled trial

## Abstract

**Background:**

We have developed a novel and brief semi-structured psychotherapeutic intervention for patients with advanced or metastatic cancer, called Managing Cancer And Living Meaningfully. We describe here the methodology of a randomized controlled trial to test the efficacy of this treatment to alleviate distress and promote well-being in this population.

**Methods/Design:**

The study is an unblinded randomized controlled trial with 2 conditions (intervention plus usual care versus usual care alone) and assessments at baseline, 3 and 6 months. The site is the Princess Margaret Cancer Centre, part of the University Health Network, in Toronto, Canada. Eligibility criteria include: ≥ 18 years of age; English fluency; no cognitive impairment; and diagnosis of advanced cancer. The 3–6 session intervention is manualized and allows for flexibility to meet individual patients’ needs. It is delivered over a 3–6 month period and provides reflective space for patients (and their primary caregivers) to address 4 main domains: symptom management and communication with health care providers; changes in self and relations with close others; sense of meaning and purpose; and the future and mortality. Usual care at the Princess Margaret Cancer Centre includes distress screening and referral as required to in-hospital psychosocial and palliative care services. The primary outcome is frequency of depressive symptoms and the primary endpoint is at 3 months. Secondary outcomes include diagnosis of major or minor depression, generalized anxiety, death anxiety, spiritual well-being, quality of life, demoralization, attachment security, posttraumatic growth, communication with partners, and satisfaction with clinical interactions.

**Discussion:**

Managing Cancer And Living Meaningfully has the potential to relieve distress and promote psychological well-being in patients with advanced cancer and their primary caregivers. This trial is being conducted to determine its benefit and inform its dissemination. The intervention has cross-national relevance and training workshops have been held thus far with clinicians from North and South America, Europe, the Middle East, Asia and Africa.

**Trial Registration:**

ClinicalTrials.gov NCT01506492 4 January 2012.

**Electronic supplementary material:**

The online version of this article (doi:10.1186/s13063-015-0811-1) contains supplementary material, which is available to authorized users.

## Background

Advanced or metastatic cancer is predictably associated with challenges and burdens that may lead to symptoms of depression and demoralization and fears of suffering, dependency, and mortality [1]. The multiple physical symptoms, the dramatic alteration in support needs and in personal relationships, the difficulty navigating a complex health care system, and the threat of impending mortality all may constitute pathways to distress in this population [2]. The challenge for individuals in this circumstance is to sustain a “double awareness” that allows them to remain engaged in life while facing the imminence of physical deterioration, shortened survival, and death [3]. A variety of individual and social factors may protect individuals in this circumstance, but professional support may also be of value to prevent and treat the distress that commonly emerges in this population [4].

Clinically significant depressive symptoms may be frequent in patients with advanced cancer and can be understood as a final common pathway of distress, emerging in response to the interaction of multiple disease-related, individual and psychosocial factors [1, 2, 5–7]. The most prominent of these are the physical burden of disease, attachment insecurity (i.e., worry about the availability of supportive relationships and the capacity to make use of them for emotional support), lower self-esteem, feelings of hopelessness and impaired spiritual well-being [1, 2]. Although many psychotherapeutic modalities have been used to treat depression (e.g., cognitive behavior therapy and interpersonal therapy), positive outcomes and sustained improvement may be most likely when treatment is directed at etiological and pathogenic factors that are specific to the context in which disturbances arise [8]. Preliminary findings in patients with advanced cancer also suggest that psychological treatments for depression are preferred over pharmacological ones [9], and that individual psychotherapy is preferred over group therapy because sessions can be flexibly tailored to patients’ individual needs, taking into account other clinic appointments and fluctuations in health status [10–13].

To address the relative lack of evidence-based individual therapies tailored for this population, we have developed a novel, brief, semi-structured, individual, manualized psychotherapeutic intervention to alleviate distress and promote well-being in patients with advanced or metastatic cancer. This psychotherapy, called Managing Cancer And Living Meaningfully (CALM) [4, 14, 15], addresses the specific problems and risk factors that contribute to the emergence of depressive symptoms in this circumstance [1, 2]. It shares features with manualized supportive-expressive [16–24], cognitive-existential [25, 26], and meaning-centered [27] group psychotherapies applied to patients with advanced and terminal disease. CALM provides support and reflective space for the processing of thoughts and emotions evoked by this traumatic condition and facilitates the resolution of practical and existential questions that face individuals with advanced disease. Such an intervention has the potential not only to relieve distress but also to promote psychological growth [28, 29]. The purpose of this phase III randomized controlled trial (RCT) is to evaluate the efficacy of the CALM intervention in patients with advanced cancer.

## Methods/Design

The study is an unblinded RCT with 2 trial conditions (intervention plus usual care versus usual care alone) and assessments at baseline, 3 and 6 months. The primary outcome is depressive symptoms as assessed by the Patient Health Questionnaire-9 (PHQ-9). The primary endpoint is at 3 months and the secondary endpoint is at 6 months. The site is the Princess Margaret Cancer Centre, part of the University Health Network and Canada’s largest comprehensive cancer treatment and research center where more than 10,000 patients are assessed annually and more than 1,000 patients attend outpatient clinics daily [30]. The phase III protocol and related documents were approved by the Research Ethics Board (REB) of the University Health Network on 5 February 2010 (UHN REB #09-0855-C). A number of subsequent amendments to allow a phase IIA non-randomized and a phase IIB randomized pilot were approved by the REB prior to the final amendment and launch of this phase III trial in January 2012. Patients are recruited from eight approved tumor sites: gastrointestinal, lung, gynecological, breast, genitourinary, sarcoma, melanoma and endocrine. Patients are approved for trial enrollment by the principal investigators (GR, SH, CL) prior to randomization.

### Inclusion/Exclusion Criteria

The inclusion criteria are: 1) ≥ 18 years of age; 2) fluency in English; 3) no cognitive impairment indicated in the medical record or by the attending oncologist; and 4) a confirmed diagnosis of stage III or IV lung cancer, any stage of pancreatic cancer (due to the aggressiveness of this disease), unresectable cholangiocarcinoma, unresectable liver cancer, unresectable ampullary or peri-ampullary cancer or other stage IV (metastatic) gastrointestinal cancer, stage III or IV ovarian and fallopian tube cancers, or other stage IV gynecological cancer; and stage IV breast, genitourinary, sarcoma, melanoma, or endocrine cancers (all of the above with expected survival of 12–18 months). Patients meeting inclusion criteria undergo a brief interview with a research staff member to identify the following exclusion criteria: 1) major communication difficulties; 2) inability to commit to the required 3–6 psychotherapy sessions (i.e., too ill to participate, lack of transportation, insufficient motivation due to lack of distress); 3) cognitive impairment as indicated by a score < 20 on the Short Orientation-Memory-Concentration (SOMC) test [31], unless deemed suitable at the recruiter’s discretion, or due to brain metastases; 4) actively receiving psychiatric or psychological intervention in the Department of Supportive Care (formerly the Department of Psychosocial Oncology and Palliative Care) at the Princess Margaret Cancer Centre at the time of study approach; 5) refusal to accept randomization; and 6) prior treatment with CALM therapy during an earlier phase of the study.

### Procedure

Research staff screen the outpatient oncology clinic lists of eight solid tumor sites on a daily basis. Patients with advanced cancer are identified and mailed introductory letters concerning the study. These patients are approached by research staff for study recruitment while attending clinic appointments. During the recruitment process, the study design and intervention are described to patients and the inclusion/exclusion criteria are reviewed. Participants are also informed at study recruitment that if they are not randomized to receive CALM therapy, they will be offered the opportunity to receive CALM after completion of the final 6-month assessment. After providing written informed consent, cognitive functioning and other exclusion criteria are assessed, medical and demographic information is collected, baseline measures are administered, a diagnostic interview for depression is conducted, and eligible patients are randomized. Some patients who screen fail may be eligible for reapproach at a later date.

### Trial conditions

Participants in the intervention group receive usual care plus CALM, a semi-structured psychotherapy designed for patients with advanced cancer. CALM was developed based on empirical data, clinical observations, and the theoretical foundations of relational [32], attachment [33] and existential [34] theory.

CALM includes 3–6 individual psychotherapy sessions, each approximately 45–60 minutes in length, delivered over 3-6 months. The sessions cover 4 domains: 1) symptom management and communication with health care providers; 2) changes in self and relations with close others; 3) sense of meaning and purpose; and 4) the future and mortality [14, 15]. All domains are addressed with each patient, but the time devoted to each and the sequence in which they are addressed is based on the salience of concerns for each patient during the session. Therapists aim to deliver at least 3 sessions within 3 months. Non-compliance with intervention is defined as having less than 3 sessions over the course of the trial.

CALM is delivered by specially trained therapists, primarily master's level social workers, in the Department of Supportive Care at the Princess Margaret Cancer Centre. Therapists are trained and supervised by the clinician investigators who developed the intervention (GR, SH). The participant’s primary caregiver (e.g., spouse/partner, adult son/daughter, family member), or significant other, is invited to participate in one or more of the therapy sessions, when acceptable to the patient.

At any time during the intervention, patients considered by the therapist to be at acute risk for suicide, or who demonstrate significant worsening of depression or other psychiatric co-morbidities that require treatment, will be referred for psychiatric assessment and treatment in the Department of Supportive Care. This may include pharmacotherapy or other psychiatric interventions.

Participants in the control group receive usual care alone. At the Princess Margaret Cancer Centre this includes routine treatment and follow-up in medical, surgical and/or radiation outpatient oncology clinics, as well as a clinic-based distress screening program, referred to as DART (Distress Assessment and Response Tool) [35], with results provided at the time of the clinic visit to oncology clinic staff. Clinic staff may refer any patient for specialized psychosocial oncology services (provided by trained volunteers and by social work, psychiatry and psychology staff) or to palliative care, based on clinical judgment, patient requests and/or distress screening scores. In the event that a usual care participant reports suicidal intent, the principal investigators are contacted and the patient is assessed by a psychiatrist from the Department of Supportive Care.

Approximately one third of patients with metastatic cancer are referred for psychosocial care at the Princess Margaret, two thirds of whom are seen by a social worker [36]. Approximately 65 % of social work consultations involve provision of practical or instrumental care (e.g., urgent drug coverage, referral for admission to the Acute Palliative Care Unit, complex continuing care placement, etc.); 35 % of consultations involve brief supportive interactions to alleviate emotional distress in patients and/or family members. Control participants do not typically receive structured counseling from social work as part of usual care. Of those referred to psychiatry or psychology, less than one third receive a structured or semi-structured psychotherapy. Overall, less than 10 % of patients with metastatic cancer at the Princess Margaret Cancer Centre receive any form of semi-structured psychotherapy that is similar to CALM therapy. To avoid contamination of the control group in the event of a referral to the Department of Supportive Care, controls referred for psychosocial care will be seen, if possible, by therapists in the department who have not received prior training in CALM therapy (i.e., non-CALM-trained therapists). Contamination will be monitored and documented and is defined as having two or more sessions with a CALM-trained therapist.

### Treatment integrity

The treatment integrity of the intervention arm is ensured by means of weekly group supervision of therapists with case presentations. CALM sessions are audio-recorded, and therapists document sessions in a written report. Senior clinicians (GR, SH) assess overall and topic-specific competencies using treatment integrity rating scales adapted from Spiegel and Spira’s [18] tools. Evaluations are discussed with each therapist to improve competencies.

### Randomization

Permuted block randomization is used to allocate participants to a trial condition with stratification by PHQ-9 score (< or ≥ 10). Stratification ensures that highly depressed individuals will be balanced in both arms. The randomization process is managed by the Department of Biostatistics at the Princess Margaret Cancer Centre, which is independent of the trial team.

### Outcome measures

The primary outcome measure is the Patient Health Questionnaire-9 (PHQ-9) [37], a reliable and valid 9-item measure of depression that has been used widely with advanced cancer patients [38]. This brief measure is a subscale of the Patient Health Questionnaire (PHQ), a patient self-report version of the Primary Care Evaluation of Mental Disorders (PRIME-MD), a widely used tool to screen for mental health disorders in primary care [39]. Two additional questions on the PHQ-9 include: question 9a which assesses suicidal intent if a patient endorses suicidal ideation (i.e., “Is there a chance you would do something to end your life?” Yes/No); and question 10 which assesses functional impairment (i.e., “How difficult have these problems made it for you to do your work, take care of things at home, or get along with other people?” Not difficult at all/Somewhat difficult/Very difficult/Extremely difficult).

The secondary outcome measures assess domains hypothesized to respond directly or indirectly to the intervention through its potential impact on communication with health care providers, shifts in personal relationships or other factors. A portion of the Mood Disorders and Optional Disorders Module from the Structured Clinical Interview for *Diagnostic and Statistical Manual of Mental Disorders* (DSM) (SCID) [40] (SCID-1 Research Version for DSM-IV-TR Axis 1 Disorders, January 2010) is administered in this study. The SCID is a semi-structured interview that allows researchers to make diagnoses of major and minor depression consistent with DSM diagnostic criteria [41].

The Generalized Anxiety Disorder-7 (GAD-7) [42] is a widely used and validated 7-item self-report measure designed to screen and assess the frequency of GAD symptoms. It is a subscale of the PRIME-MD [39]. An eighth item, rating how difficult these symptoms have made it to do work, take care of things at home, or get along with other people, has been included.

The Functional Assessment of Chronic Illness Therapy-Spiritual Well-being Scale (FACIT-Sp) [43] is a 12-item self-report measure of spiritual well-being, assessing the sense of meaning, peace and faith, that has been widely used in palliative care research [44, 45].

The Posttraumatic Growth Inventory (PTGI) [46–48], a 21-item self-report scale that measures positive psychological changes after trauma, has been used as a measure of psychological growth in response to the psychological trauma of cancer [49] and as an outcome measure for intervention studies [50, 51]. The PTGI provides a total score based on the experience of new possibilities and spiritual change, growth in personal strength, relations with others, and appreciation of life.

The Quality of Life at the End of Life-Cancer Scale (QUAL-EC) [52] is a measure of quality of life in patient populations near the end of life. We omitted the symptom control subscale, thereby reducing this self-report measure to 14 items.

Death anxiety is assessed using the 15-item Death and Dying Distress Scale (DADDS), which we have developed for use in advanced cancer [53]. Unlike other death anxiety measures (e.g., [54]), the DADDS is designed for populations facing imminent death. It addresses fears about the dying process, and about lost opportunities and self-perceived burden placed on others as a result of impending mortality.

The Demoralization Scale (DS) [55] is a 24-item self-report measure that assesses loss of meaning and purpose, disheartenment, and helplessness.

Attachment security is assessed using the 16-item modified Experiences in Close Relationships Inventory (ECR-M16) [56], tailored for use with advanced cancer patients. It assesses attachment avoidance (i.e., discomfort with closeness and discomfort depending on others) and attachment anxiety (i.e., fear of rejection or abandonment).

Participants who are married, common-law, or in a long-term relationship will be asked to complete the ten-item Couple Communication Scale (CCS) [57], which is concerned with an individual’s feelings, beliefs, and attitudes about the communication in his/her relationship; the CCS is taken from the PREPARE/ENRICH Inventory [57].

Lastly, the Clinical Evaluation Questionnaire (CEQ) is a seven-item measure that we have newly developed to assess the extent to which individuals feel emotionally supported by clinical services in the domains relevant to CALM therapy. For intervention participants, the CEQ refers to the patients’ experience of CALM therapy. For control participants, the CEQ refers to the patient’s interactions with the health care team at the Princess Margaret. The CEQ is assessed only at 3 and 6 months. See Additional file 1 for this measure.

Additional data collected will include: demographics, medical and psychiatric history, performance status, and disease-related symptom severity. Performance status is rated by research staff with patient input at all study time points using the Karnofsky Performance Status (KPS) scale [58]. A shortened version of the Memorial Symptom Assessment Scale (MSAS) [59] is used to measure the presence and severity of 28 common physical symptoms of cancer.

### Initial power calculations

Although the primary endpoint was designated at 3 months, sample size calculations took into account the secondary 6-month endpoint in order to sufficiently power the trial to examine outcomes at study end. We used the following sample size formula for an analysis of covariance (ANCOVA) design in which two groups are compared at follow-up, controlling for baseline scores [60]:

$$ \mathrm{n} = \left[2{\left({\mathrm{Z}}_{\mathrm{A}} + {\mathrm{Z}}_{\mathrm{B}}\right)}^2\left(1\ \hbox{--}\ {\mathrm{r}}^2\right)/{\mathrm{d}}^2\right] + 1 $$

where d = (X̅_1_ – X̅_2_)/SD, i.e., Cohen’s d [61];

n = sample size per treatment group required at follow-up;

Z_A_ = 1.96, the z-score associated with a two tailed test at alpha 0.05;

Z_B_ = 0.842, the z-score associated with a desired power of 0.80; and

r = correlation between measurements at baseline and study end.

Based on this longitudinal study: [61] (CIHR #MOP 62861) of metastatic gastrointestinal and lung cancer patients [1, 2], we observed a correlation of 0.72, n = 137, between depression scores at baseline and 6 months. We used 0.70 as our estimate of r. We planned to detect d = 0.405, a small to medium sized effect [61], consistent with prior work [9, 62].

Substituting these values into the equation results in:

$$ \mathrm{n} = \left[2{\left(1.96+0.842\right)}^2\left(1\hbox{--} 0.7{0}^2\right)/0.40{5}^2\right]+1=\left[2(7.851)(0.51)/(0.164)\right]+1=49.8 \sim 50 $$

A minimum of 50 participants per group was initially required at study end.

The following formula was used to adjust for attrition and non-compliance with intervention (i.e., having less than three CALM sessions) [63, 64]:

$$ {\mathrm{n}}_{\mathrm{b}} = {\mathrm{n}}_{\mathrm{e}}\left(1/\mathrm{p}\right)\ \left(1/{\mathrm{c}}^2\right) $$

where n_b_ = sample size required at baseline per treatment group;

n_e_ = sample size required at endpoint per treatment group;

p = proportion of participants who will reach study end; and

c = proportion of participants compliant with intervention.

We initially estimated a trial completion rate of 60 % and compliance rate of 80 % based on prior research [38]. Substituting relevant values into the equation results in:

$$ {\mathrm{n}}_{\mathrm{b}}=50\left(1/0.60\right)\left(1/0.8{0}^2\right)=50(1.667)(1.563)=130.3 \sim 131 $$

Therefore, 131 participants per group or 262 total participants will be required at baseline. Based on previous experience [1, 2], trial recruitment was anticipated to last 4.5 years.

### Sample size recalculation

A sample size recalculation was conducted in February 2014 in light of observed differences from initial estimates in rates of attrition and compliance. This procedure was undertaken with no awareness or examination of treatment effects. The observed correlation between depression scores at baseline and 6 months was 0.50, n = 112. To detect an effect size of 0.405 with a 2-tailed test, the required n per group is 73 at study end. Approximately 75 % of participants had reached study end at 6 months and 90 % of participants were compliant with intervention. Adjusting for these factors, a minimum of 121 participants per group or 242 participants overall will be required at baseline. Note that contamination of control participants (i.e., having two or more sessions with a CALM-trained therapist) was negligible and was not adjusted for.

### Proposed analyses

Analyses will be by intention to treat. ANCOVA will be used to test for outcome differences between experimental and control groups at follow-up, controlling for baseline scores and covariates, specifically age, gender and symptom burden from disease. Sensitivity analyses, including complete case analysis and multiple imputation, will be conducted to assess the impact of missing values. Linear mixed effects modeling will be used to test for group differences in trajectory over time. Intervention participants are expected to show greater benefit (i.e., less distress or greater well-being) over time relative to control participants. Structural equation modeling and factor analysis may also be used to study treatment effects on combined or composite outcomes.

The outcome of death anxiety may require special consideration, since death anxiety scores at baseline in the very low range can represent minimization or non-reflectiveness about such concerns (unpublished observations). Non-reflective individuals may increase in death anxiety as their disease progresses and as avoidant psychological strategies become less effective with physical decline. Analyses will, therefore, examine the effect of removing individuals with low death anxiety scores at baseline (i.e., DADDS < 15). Effective psychotherapeutic intervention may actually be associated with increased death anxiety due to the processing of such concerns, thereby weakening the power to detect significant treatment effects.

## Discussion

CALM therapy is a novel psychotherapeutic intervention that we have developed to alleviate distress and to promote psychological growth and well-being in patients with advanced or metastatic cancer. Unique features of this supportive-expressive therapy include its theoretical foundation in relational, attachment and existential theory, its tailored focus on the problems of advanced cancer, the inclusion of primary informal caregivers in one or more sessions of the intervention, and the potential for recruitment of participants from a clinic-based population. Although depression is specified as the primary outcome, we also place great importance on the secondary outcome measures, which we believe are highly relevant to the problem of advanced cancer and may be improved by the intervention.

There are particular challenges in an RCT of this kind. These include the identification of participants motivated to engage in psychotherapy at the time of recruitment, recruitment and retention of patients with advanced illness, and the possibility that outcomes will be affected by dramatic changes in clinical status that may occur during the course of advanced cancer. The clinic-based recruitment strategy is facilitated by the engagement of the research team with the oncology clinic staff. This clinic-based approach will be of great value to inform our understanding of the benefit of CALM in both depressed and non-depressed individuals who were not necessarily seeking professional psychosocial care.

We are fortunate to have recruited a group of committed and experienced psychosocial clinicians, who have been intensively trained and supervised to deliver the CALM intervention. Preliminary qualitative [4] and quantitative pilot studies [65] have provided support for the feasibility and value of CALM therapy, but the large phase III RCT described here will provide a more definitive assessment of its potential efficacy. Such evidence will determine whether it can be justified to routinely incorporate CALM into the comprehensive care of patients with advanced cancer.

## Trial status

The trial is currently underway. Trial Registration: ClinicalTrials.gov NCT01506492.

## Additional file

Additional file 1:
**The Clinical Evaluation Questionnaire (CEQ)*.**

## Abbreviations

ANCOVA: analysis of covariance
CALM: Managing Cancer And Living Meaningfully
CCS: Couple Communication Scale
CEQ: Clinical Evaluation Questionnaire
DADDS: Death and Dying Distress Scale
DART: Distress Assessment and Response Tool
DS: Demoralization Scale
DSM: *Diagnostic and Statistical Manual of Mental Disorders*
ECR-M16: 16-item modified Experiences in Close Relationships Inventory
FACIT-Sp: Functional Assessment of Chronic Illness Therapy-Spiritual Well-being Scale
GAD-7: Generalized Anxiety Disorder-7
KPS: Karnofsky Performance Status
MSAS: Memorial Symptom Assessment Scale
PHQ: Patient Health Questionnaire
PHQ-9: Patient Health Questionnaire-9
PRIME-MD: Primary Care Evaluation of Mental Disorders
PTGI: Posttraumatic Growth Inventory
QUAL-EC: Quality of Life at the End of Life-Cancer Scale
RCT: randomized controlled trial
REB: Research Ethics Board
SCID: Structured Clinical Interview for DSM Diagnoses
SOMC: Short Orientation-Memory-Concentration test
UHN: University Health Network
