# Supplementary material for: Managing Cancer And Living Meaningfully: study protocol for a randomized controlled trial
Source: Trials. 2015 Sep 3;16:391. doi: 10.1186/s13063-015-0811-1 (PMC4557481; doi:10.1186/s13063-015-0811-1)
Supplement: Additional file 1: — The Clinical Evaluation Questionnaire (CEQ)*. [file 13063_2015_811_MOESM1_ESM.doc]

**Additional file 1.** The Clinical Evaluation Questionnaire (CEQ)*

Please take a moment to think about your [*CALM therapy sessions* OR *interactions with the health care team at Princess Margaret Hospital*]. If an item does not apply to you, please put an “X” in the box marked “N/A.” Please put an “X” in only one box per line.

N/A – Not applicable

0 – Not at all

1 – A little bit

2 – Somewhat

3 – Quite a bit

4 – Very much

|  | To what extent [*has your CALM therapy* OR *have your interactions with the health care team at Princess Margaret Hospital*] helped you to: | N/A | 0 | 1 | 2 | 3 | 4 |
| --- | --- | --- | --- | --- | --- | --- | --- |
| 1. Freely discuss my concerns about cancer and my treatment options | |  |  |  |  |  |  |
| 1. Talk and feel understood about how cancer has affected my life | |  |  |  |  |  |  |
| 1. Deal with changes in my relationships as a result of cancer | |  |  |  |  |  |  |
| 1. Explore better ways to communicate with my health care team, my family and others | |  |  |  |  |  |  |
| 1. Clarify my values and beliefs | |  |  |  |  |  |  |
| 1. Talk about my concerns about the future and to be less frightened | |  |  |  |  |  |  |
| 1. Better express and manage my feelings | |  |  |  |  |  |  |

Please feel free to share any comments (positive or negative) about your [*CALM therapy* OR *interactions with the health care team at Princess Margaret Hospital*]:

*Note. The CEQ is worded differently depending on whether a control or intervention participant is completing the form, as indicated by the brackets. For intervention participants, the questionnaire refers to CALM therapy. For control participants, the questionnaire refers to interactions with their health care team at Princess Margaret Hospital.
